# Is there an advantageous arrangement of aromatic residues in proteins? Statistical analysis of aromatic interactions in globular proteins

**DOI:** 10.1016/j.csbj.2021.10.036

**Published:** 2021-11-01

**Authors:** Mikhail Yu. Lobanov, Leonid B. Pereyaslavets, Ilya V. Likhachev, Bakhyt T. Matkarimov, Oxana V. Galzitskaya

**Affiliations:** a Institute of Protein Research, Russian Academy of Sciences, 142290 Pushchino, Moscow Region, Russia; b Institute of Mathematical Problems of Biology, Russian Academy of Sciences, Keldysh Institute of Applied Mathematics, Russian Academy of Sciences, 142290 Pushchino, Moscow Region, Russia; c National Laboratory Astana, Nazarbayev University, Nur-Sultan, Kazakhstan; d Institute of Theoretical and Experimental Biophysics, Russian Academy of Sciences, 142290 Pushchino, Moscow Region, Russia

**Keywords:** Aromatic residues, Conformation, Exhaustive enumeration, Stacking, Server, Protein Data Bank, Identity

## Abstract

The aim of this study was to evaluate the favorability of different conformations of aromatic residues in proteins by analysing the occurrence of particular conformations. The clustering of protein structures from the Protein Data Bank (PDB) was performed. Conformations of interacting aromatic residues were analyzed for 511 282 pairs in 35 493 protein structures sharing less than 50% identity. Pairs with a parallel arrangement of aromatic residues made up 6.2% of all possible ones, which was twice as much as expected. Pairs with a perpendicular arrangement of aromatic residues made up 25%. We demonstrate that the most favorable arrangement was at an angle of 60° between the interacting aromatic residues. Among all possible aromatic pairs, the His-His pair was twice as frequent as expected, and the His-Phe pair was less frequent than expected. A server (CARP – Contacts of Aromatic Residues in Proteins) has been created for calculating essential structural features of interacting aromatic residues in proteins: http://bioproteom.protres.ru/arom_q_prog/.

## Graphical abstract

**Figure:**
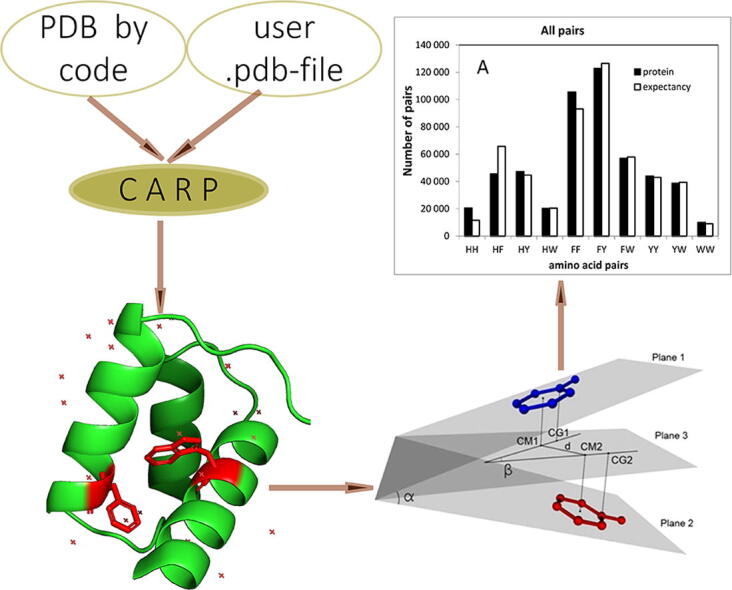

## 1 Introduction

The interaction of π-resonance structures plays an essential role in the stability of biological macromolecules such as proteins and DNA. The main features of π-resonance structures are their relatively large sizes, the rigidity of π-orbitals, quadrupole momentum, and dispersion. Among the most common π-resonance structures are aromatic rings. The stacking of nucleotides plays a key role in DNA stabilization. The aromatic stacking effect in proteins is less obvious. In protein molecules, several amino acid residues are the frequent participants in the aromatic interaction; the conception of such aromatic interaction can be found in reviews [1], [2], [3].

Studies of aromatic interactions made it possible to expand our knowledge of the contribution of these residues to the stability of protein structures. Burley and Petsko were the first to calculate the distributions of aromatic-aromatic and amino-aromatic interactions in proteins [4], [5]. McGaughey et al. performed a detailed aromatic-aromatic investigation for a set of 505 proteins. They evaluated the energetic difference between T-stacking (perpendicular stacking) and PD-stacking (parallel displaced stacking) of all aromatic structures [6]. The results of further studies showed a significant effect of aromatic clusters upon macroscopic thermophilic properties [7]. Aromatic interactions in α-helices were studied and many dependencies of close-to-sequence interactions were revealed [8]. Concurrent with these studies, Thomas et al. researched and described an almost consistent aromatic-aromatic interaction [9], [10], [11], [12]. They showed that the binding of aromatic residues on the long-range sequence is 74% of all pairs, and that mostly two β-strands were linked. After Chipot et al. [13] investigated the free energy interaction of Phe-Phe in an aqueous medium, Chelli et al. studied aromatic dimers Phe-Phe, Phe-Tyr and Tyr-Tyr by simulating these dimers in various solvents, and strengthened their findings through the statistical analysis on a set of two thousand proteins [14].

It has been demonstrated that aromatic interactions can initiate the disorder-to-order transitions in the intrinsically disordered regions of proteins [15]. Stacking interactions are essential for the stability of macromolecular complexes. Many nucleoproteins use aromatic stacking to recognize a binding site on DNA or RNA. Fundamental processes such as mismatch repair, strand separation, deadenylation, degradation, and RNA cap-binding involve stacking interactions. Compounds that can block stacking interactions could potentially be of pharmaceutical importance [16].

In this work, we performed statistical analysis of aromatic residues interactions (511 282 pairs) in the database of 35 493 protein structures with the identity below 50% from the clustered Protein Data Bank (PDB), in order to determine a favorable packing of aromatic residues in the proteins. The data obtained can be found at http://bioproteom.protres.ru/arom_q_prog/. This server allows the analysis of any protein structure using the PDB format.

## 2 Materials and methods

### 2.1 Construction of clustered Protein Data Bank

The PDB clustering process had to be carried out as to take into account closely related structures together: in order to consider one representative [17]. For example, if we consider the spatial structures of proteins, it is more convenient to select representative ones, as it will be done in the case of analysis of aromatic residues in proteins. However, for disordered residues it is better to take the entire set of chains with different weights.

The database was generated from the PDB dated December 6, 2020. Only structures solved by X-ray diffraction analysis with resolution better than 3 Å and the length of ≥40 residues were taken into account. A total of 321 545 chains were selected, comprising 89 538 846 residues. Perfectly matched chains were combined into a 100% identity group of 85 013 chains comprising 23 869 974 residues. Grouped chains (C100) were clustered together with an identity of *Id* ≥ 75% (C75, between any pair of chains in the cluster, *Id* ≥ 75%). The BLAST program was used for alignment; identity calculated using the equation below:

(1) $$\mathrm{Id}=\frac{I}{L_{1}+L_{2}-I}\times 100\%,$$

where *I* is the number of identical residues, *L_1_* and *L_2_* are the numbers of amino acid residues in each considered protein.

**Fig. 1:**
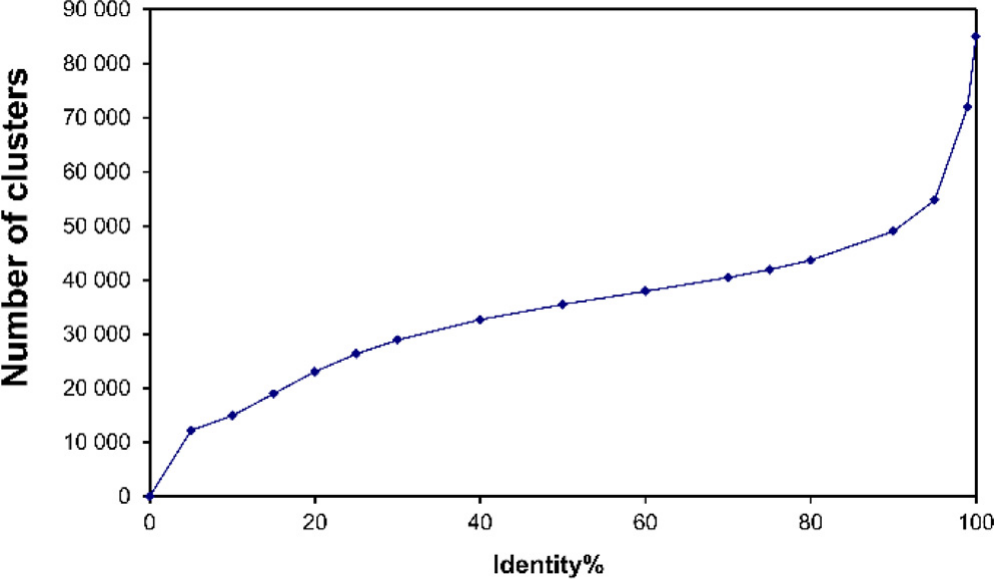
Dependence of the number of clusters on the identity of protein chains for the clustered PDB (version 2020). Within the cluster Id ≥ 75%, all sequences have an identity above 75%. The main feature of our clusters is that a cluster with Id ≥ 50% includes clusters with Id ≥ 75% as a whole.

The clustering procedure involved combining a pair of chains with the maximum *Id*, then another pair of chains or chains with a cluster, again with a maximum *Id*, etc. In the case of association of a chain with a cluster, or a union of clusters the average *Id* of the association was considered. If the *Id* of at least a pair of chains from different clusters was less than 75%, the clusters were not combined. The operation was repeated for as long as there were clusters that could be combined. Clusters C50, C25, and C05 were obtained similarly. The dependence of the number of clusters on the identity of chains within a cluster is shown in Fig. 1. In total, the following numbers of clusters were obtained: C75 – 41 925 clusters; C50 – 35 493 clusters; C25 – 26 330 clusters; C05 – 12 193 clusters. The structure of highest resolution and with fewest errors was selected from a group of C50. More information can be found at the website http://bioproteom.protres.ru/cluster_pdb/.

### 2.2 Enumeration of all possible conformations for a pair of aromatic residues

To study the aromatic rings arrangements in proteins a featureless distribution is required as a starting point, which in what follows, we will call the reference distribution. To obtain this, we considered all possible arrangements for a pair of phenylalanine aromatic rings. All possible conformations of two aromatic rings were enumerated as follows. To begin with, we took the aromatic group of phenylalanine and shifted it so that the center of the aromatic group was in the center of coordinates. Then, we obtained the position of the second aromatic group of phenylalanine using the rotational matrix *m* and the shift vector *v*. Since the center of the aromatic group coincided with the center of coordinates, the use of a rotation matrix did not change the distance between the centers of the pair of aromatic groups. This distance was determined entirely by the vector *v* and is equal to the length of this vector. Thus, to enumerate all possible mutual conformations, it is necessary to enumerate all possible *m* and *v*.

Since we determined that contact between a pair of aromatic residues exists if the distance between the centers of the aromatic rings is less than 7 Å, it was not at all difficult to enumerate all possible *v*. We ran over all possible values of x, y, and z from −7 Å to 7 Å with a step of 0.5 Å.

Sorting the rotation matrices was more difficult. The simplest solution is to take all rotation matrices around the 0X axis with a given step, similarly with 0Y and 0Z. The resulting matrix is obtained by multiplying these three. Unfortunately, this method provides too many matrices close to each other, which are not evenly distributed. Therefore, we set the task to create a set of uniformly distributed matrices. To do this, we took an uneven set obtained by rotating around the 0X, 0Y, and 0Z axes with a step of 1° and sifted it, removing the matrices which were too similar.

**Fig. 2:**
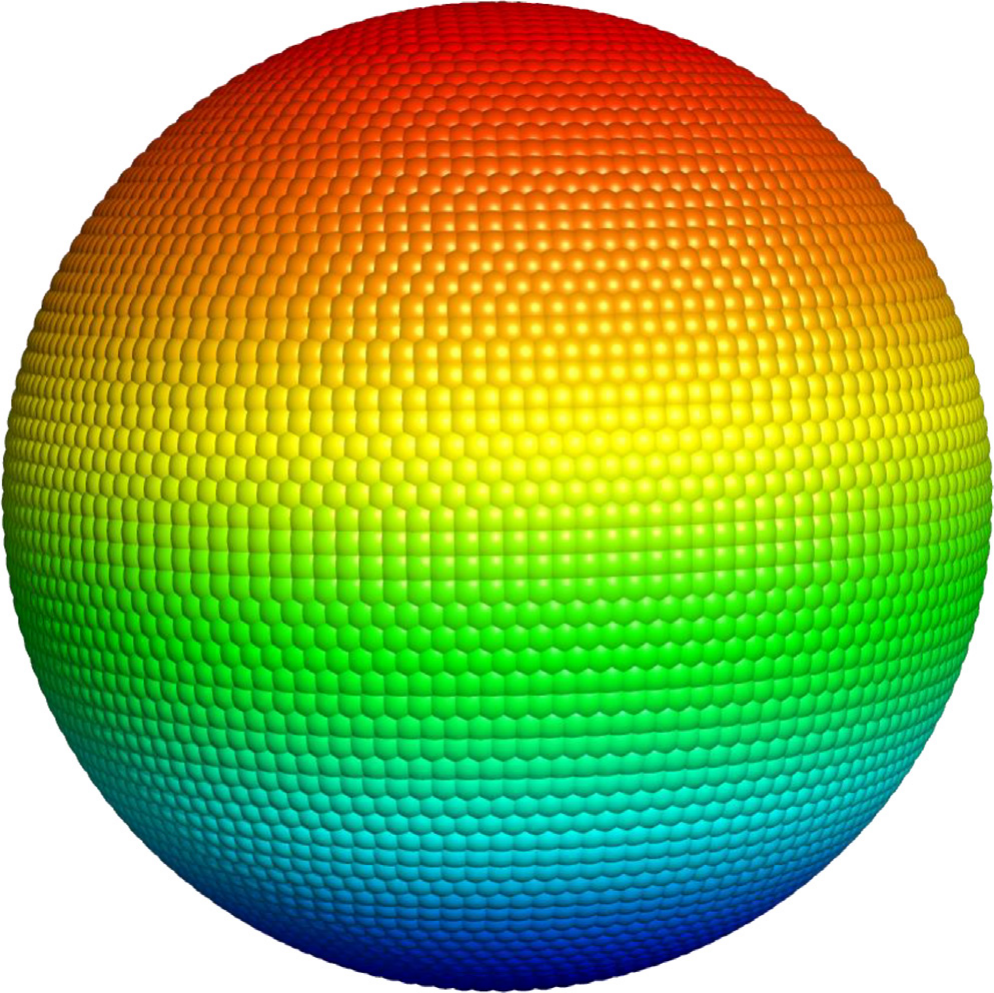
Sphere with points *P* uniformly distributed on it.

To remove matrices evenly, we placed 6600 points on a sphere of unit radius. The angle between the nearest points *P* and the center of the sphere C ∠P_1_CP_2_ = 2.5°. For each rotation matrix ***m_i_***, we define vectors: ***x_i_*** = {1,0,0}∙***m_i_***, ***y_i_*** = {0,1,0}∙***m_i_***, ***z_i_*** = {0,0,1}∙***m_i_***. The point *P* closest to *x_i_* is called *P_xi_*. We define *P_yi_* and *P_zi_* in a similar way. All rotation matrices ***m*** were found for which *P_xi_*, *P_yi_,* and *P_zi_* coincide. We kept only one of them, for which the distance $D={\left({\left(x_{i}-P_{\mathrm{xi}}\right)}^{2}+{\left(y_{i}-P_{\mathrm{yi}}\right)}^{2}{+\left(z_{i}-P_{\mathrm{zi}}\right)}^{2}\right)}^{1/2}$ was minimal. As a result of selection, 833 201 rotation matrices were obtained. In total, 9.5*10^9^ conformations were considered. Points *P* are shown in Fig. 2. A set of rotation matrices is available at http://bioproteom.protres.ru/arom_q_prog/images/an_360_72.zip.

Subsequently, we used one matrix with the initial coordinates and the other in one of the obtained 9.5*10^9^ conformations. Therefore, of these aromatic pairs, 0.34*10^9^ are parallel and 2.5*10^9^ are perpendicular (Table 1).

**Table 1:** The number of possible conformations of aromatic pairs in a complete enumeration.

	Exhaustive search with overlapping	Exhaustive search with non-overlapping	Proteins
All	9.57 × 10^9^ (100%)	2.97 × 10^9^ (100%)	4.29 × 10^5^ (100%)
α < 15° or α ≥ 165°(Parallel)	0.34 × 10^9^ (4%)	0.12 × 10^9^ (4%)	0.27 × 10^5^ (6%)
15° ≤ α < 45°	2.06 × 10^9^ (22%)	0.74 × 10^9^ (25%)	1.07 × 10^5^ (25%)
45° ≤ α < 75°	2.79 × 10^9^ (29%)	0.97 × 109 (33%)	1.28 × 10^5^ (30%)
75° ≤ α < 105° (Perpendicular)	2.50 × 10^9^ (26%)	0.75 × 10^9^ (25%)	1.07 × 10^5^ (25%)
105° ≤ α < 135°	1.42 × 10^9^ (15%)	0.32 × 10^9^ (11%)	0.51 × 10^5^ (12%)
135° ≤ α < 165°	0.45 × 10^9^ (5%)	0.07 × 10^9^ (2%)	0.10 × 10^5^ (2%)

It is clear that among the aromatic pairs obtained there were pairs with overlapping atoms. We considered all possible conformations, as well as non-overlapping conformations. In the latter case, the main chain was modeled by a ball with a radius of 3 Å, the center of which was located on the C_γ_-C_β_ line and was located at a distance of 3 Å from C_β_. Clearly, should we have not chosen the ball, we would need to add rotation at two angles for each of the residues. Each atom of the side chain was modeled by a ball with a radius of 1.75 Å. Conformations with overlapping balls were prohibited. 3.0*10^9^ non-overlapping pairs were obtained. Of these, 0.12*10^9^ are parallel and 0.75*10^9^ are perpendicular pairs. Interestingly, parallel and perpendicular pairs were eliminated in almost the same way. After the selection there was only a third of the pairs left.

## 3 Results and discussion

### 3.1 Analysis of aromatic residues in the clustered Protein Data Bank

**Fig. 3:**
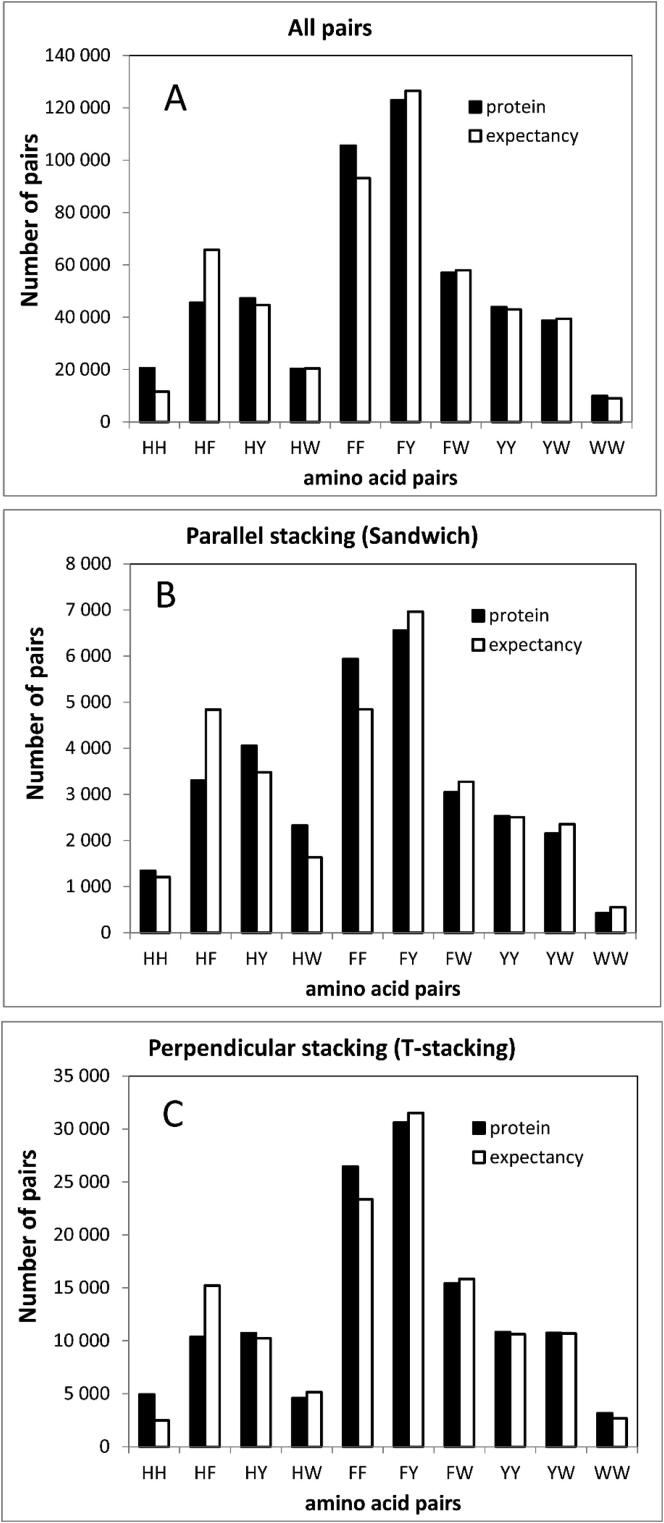
The number of pairs of aromatic residues in the studied set of proteins (35 493 structures) and the expected number of pairs from the product of probabilities. N_exp H-F _ = 2*N_sum_*P_H_*P_F,_ where P_H_ and P_F_ are the frequencies of histidine and phenylalanine occurrences in the aromatic pairs, N_sum_ is the number of aromatic pairs. (A) for all pairs; (B) for parallel staking; (C) for perpendicular stacking.

In this work, 35 493 protein structures from PDB with less than 50% identity were analyzed. This identity threshold was chosen because it was sufficient to exclude fairly similar proteins and in doing so to obtain a nonredundant database. We investigated the interaction between the four aromatic amino acid residues (Phe, Tyr, Trp, and His). For further analysis, all pairs with a distance between the centers of the aromatic groups less than 7 Å, separated by more than one residue along the chain, were extracted from the proteins to avoid steric restrictions. The number of pairs with centers of the aromatic groups closer than 7 Å is shown in Fig. 3A.

**Fig. 4:**
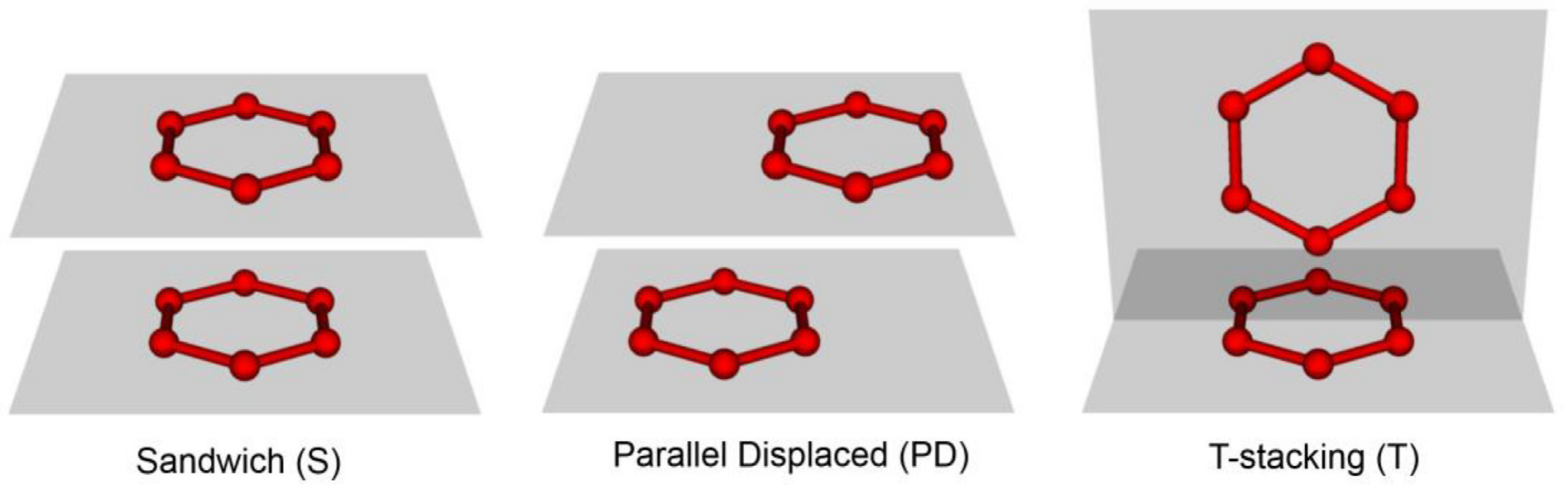
Conformations of aromatic rings under consideration.

Since the strict parallelism and the strict perpendicularity are not observed in proteins, we considered the aromatic rings to be parallel if the angle between the planes was in the range from 0° to 15°, or in the range from 165° to 180° (Fig. 4). Likewise, aromatic rings are perpendicular (T-stacking) if the angle between the planes is in the range of 75° to 105°. A total of 511 282 aromatic pairs were found in the proteins. Of these, 31 658 are parallel, and 127 788 are perpendicular pairs (Fig. 3B, C).

### 3.2 Analysis of conformations for interacting aromatic residues

The first interesting result concerns the number of aromatic pairs. Within our database of 35 493 proteins, parallel pairs were making up 6.2% of all possible ones. At the same time, a complete enumeration of all possible conformations for a pair of aromatic residues gave 3.6% of parallel ones, while among the pairs without overlapping this was 3.9% (Table 1). The fraction of parallel pairs in the proteins was therefore almost twice as high compared to the purely steric expectations (i.e. the mathematical expectation built without considering the interaction of aromatic groups, except for the prohibition by overlapping of atoms). Undoubtedly, this must happen due to the stacking interactions of aromatic rings. But, on the other hand, the benefit from the parallel arrangement is not so great as to turn around the aromatic rings that exist in proteins at an angle of 30°.

At the same time, the fraction of perpendicular aromatic rings is 25% both for the clustered proteins and the reference database (Table 1). This means, perpendicular stacking is energetically neutral.

It should be noted here that the ideal medium for simulating the interaction of aromatic rings in proteins would be a solution of benzene in octane. Aromatic groups in proteins are located within hydrophobic environment of the core. Therefore, no great energetic advantage is gained from the contact of aromatic rings with each other compared to what would be gained from their contacts with aliphatic hydrophobic groups. This is most likely why we observe such a variety of conformations.

Let us consider the occurrence of individual aromatic pairs. It turns out that the His-His pair is found in the clustered proteins twice as often as the expected frequency of the pair calculated from the probabilities of histidine occurrence. In addition, this was prevalently due to the presence of the perpendicular pairs. In contrast, the occurrence of the His-Phe pair was found to be below the expected value (Fig. 3).

**Fig. 5:**
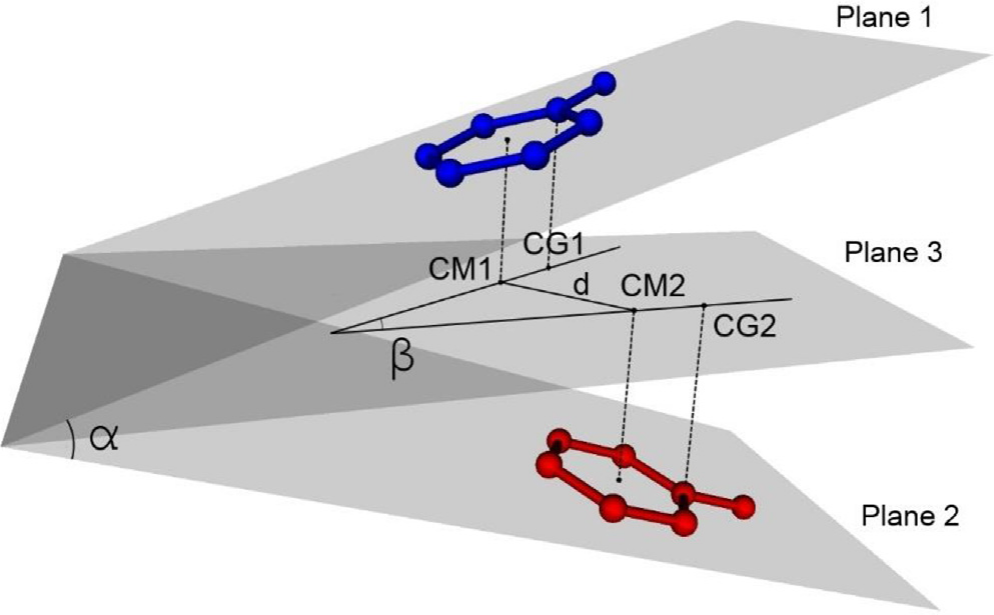
Schematic representation of the parameters calculated for a pair of aromatic residues: α is the angle between plane 1 and plane 2, β is the angle between the aromatic rings, and d is the shift between the centers of the aromatic groups projected on the central plane 3. Plane 3 divides the dihedral angle in half.

We further analyzed the three main parameters (Fig. 5): the angle between the planes of the aromatic groups, α; the angle between the guiding lines of the side groups of aromatic amino acids i.e. the angle between the vectors formed by the centers of the aromatic groups and the C_γ_ atom (for tyrosine and phenylalanine, this direction coincides precisely with the C_γ_-C_β_ bond), β; as well as the distance between the guiding lines of aromatic rings, *d*. To determine the last two characteristics, all projections were made to the plane passing through the intersection of two planes where the two groups lie, and the point corresponding to the middle of the line between the centers of aromatic rings.

The α angle was introduced to distinguish between parallel (0°, 180°) and perpendicular (90°) conformations (interplanar angle), the β angle to separate cis-, trans- and intermediate conformations, and a shift to assess the level of shielding of each of them by aromatic rings. For parallel contact, the latter parameter means the shift between the centers of the aromatic rings.

**Fig. 6:**
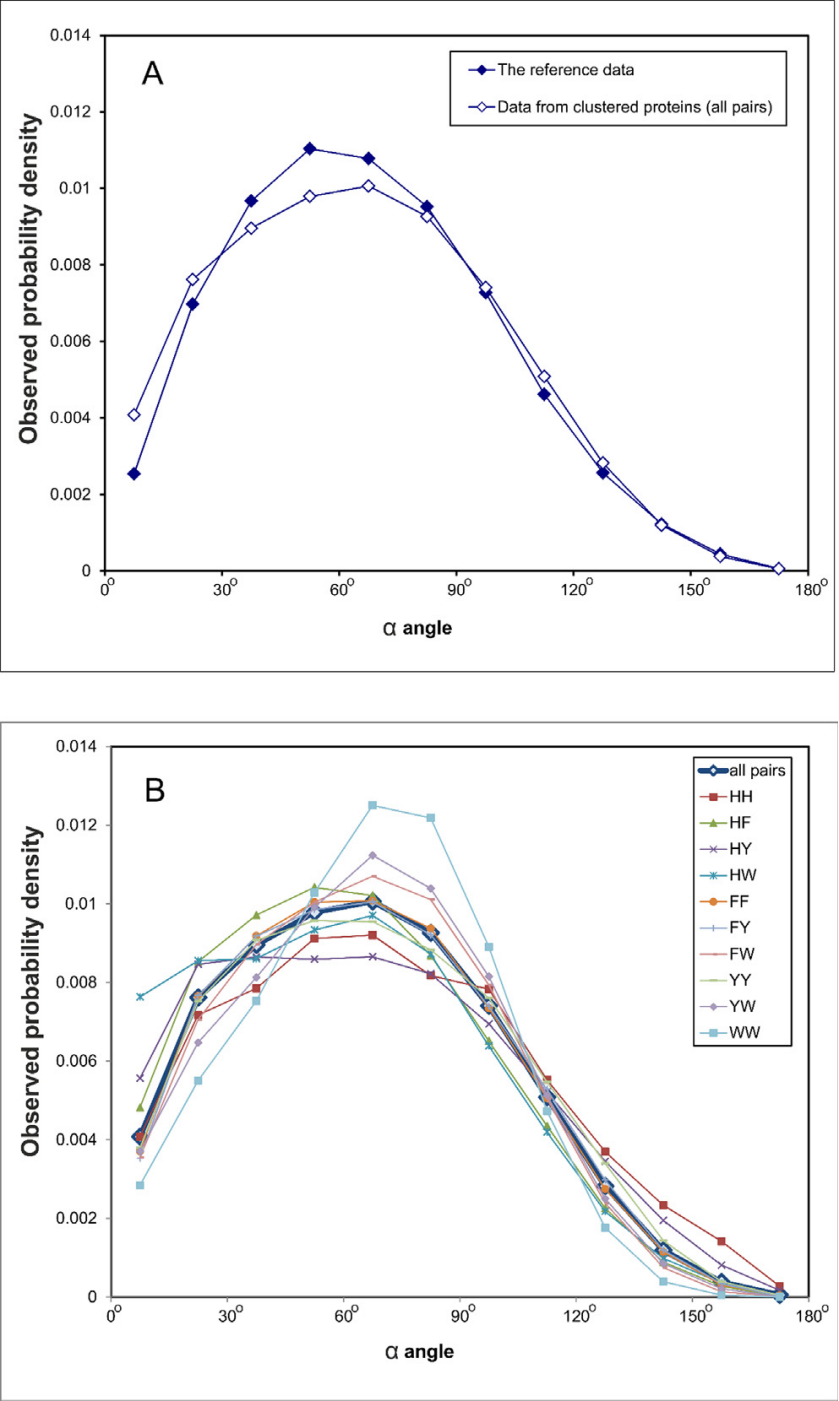
α Angle distribution between planes for (A) all interacting aromatic residues and (B) for various aromatic pairs. The observed probability density is calculated with the expression: Ni/(N*Δ) where Ni is the number of pairs in the interval, N is the total number of pairs, and Δ is the width of the interval. The reference data were obtained with a complete enumeration of all non-overlapping conformations of aromatic pairs.

We compare the data obtained for proteins against the reference data for pairs with non-overlapping atoms. However, the difference appeared to be small and statistically insignificant. Note the distribution of an angle α (Fig. 6).

We determined the experimental probability density as the fraction of residues in a given interval, normalized to the width of the interval. This measure allows to display distributions with very different amounts of data on one graph. In accordance with the general considerations, we obtained a typical sinusoidal distribution, as explained in the next section. Since we consider only contacting aromatic residues, the maximum is displaced towards acute angles and is located at an angle of 60°. However, if the aromatic groups' centers coincided, we would have a strictly sinusoidal distribution. But this is not possible due to steric prohibitions. Here we see that there are more parallel pairs in the proteins. However, this effect is not very significant.

**Fig. 7:**
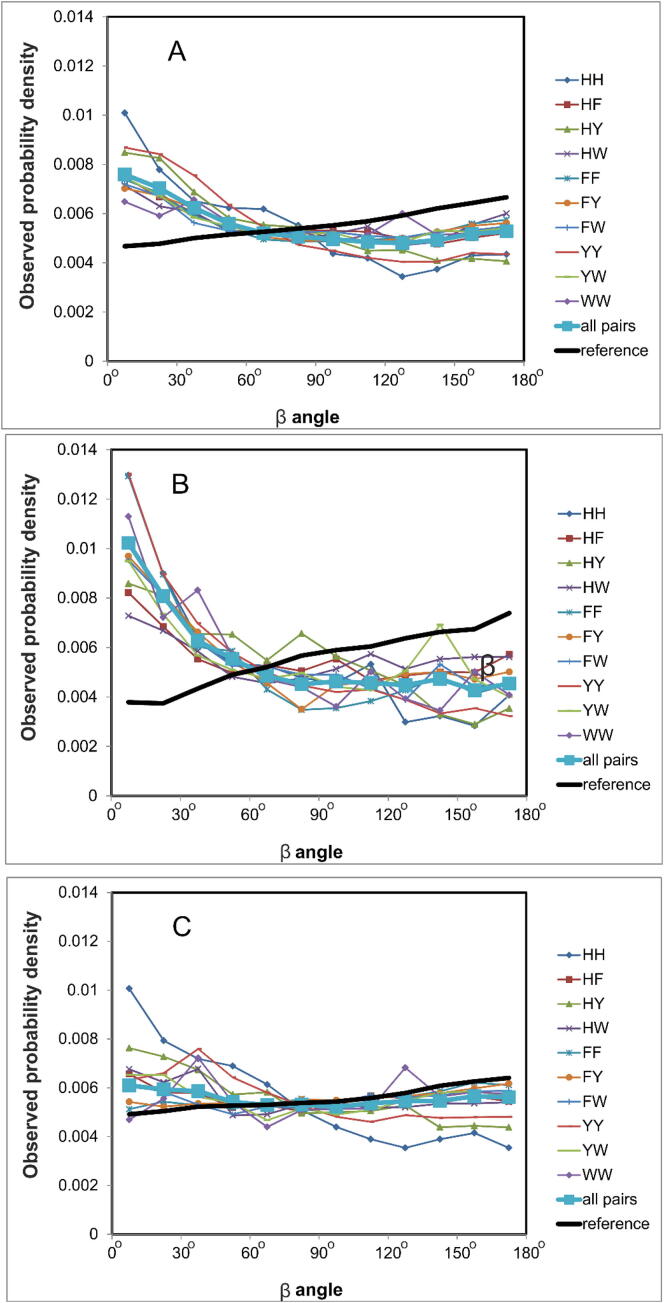
β Angle distribution between interacting aromatic residues. (A) for all pairs; (B) for parallel staking; (C) for perpendicular stacking. The reference distribution was obtained for a complete enumeration of all non-overlapping conformations of aromatic pairs.

**Fig. 8:**
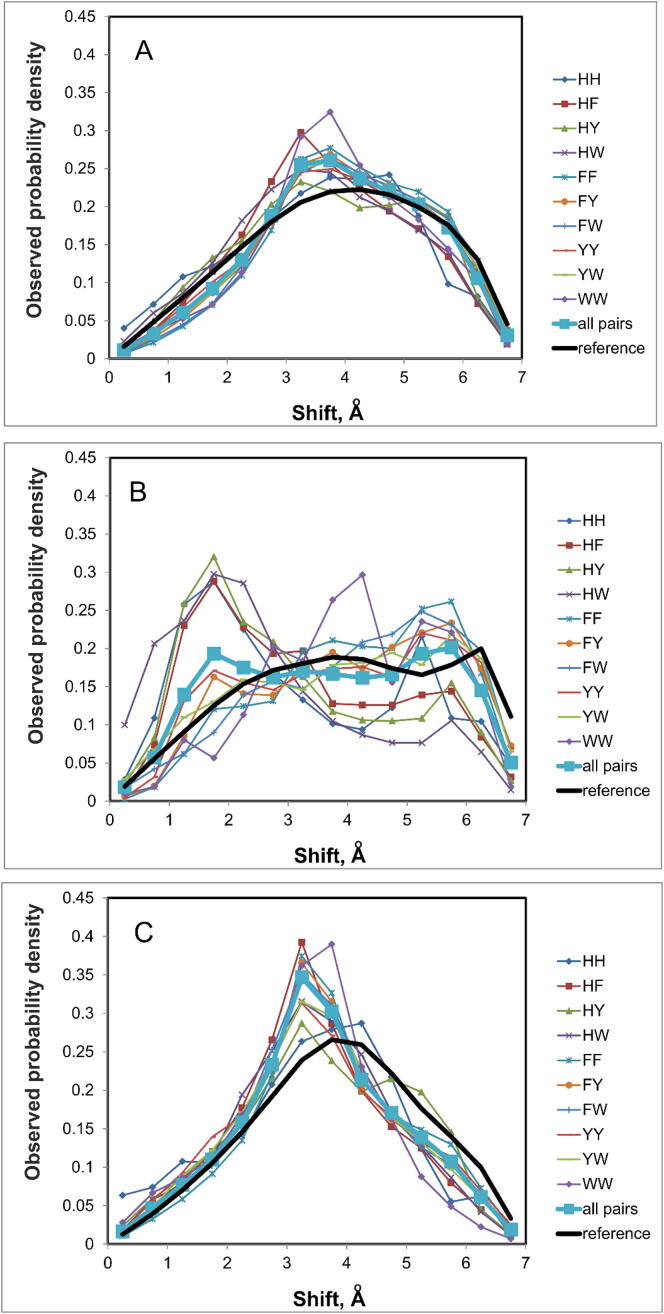
Shift distribution. For parallel contact this parameter (shift) means the shift between the centers of the aromatic rings. (A) for all pairs; (B) for parallel staking; (C) for perpendicular stacking. The reference distribution was obtained for a complete enumeration of all non-overlapping conformations of aromatic pairs.

The distribution of β rotation angle of aromatic residues is close to uniform (Fig. 7). Interestingly, the greatest deviations from the uniform distribution are found for the parallel pairs. In this case, the complete enumeration of all possible conformations for a pair of aromatic residues gave trans-positions (180°) predominantly, while cis-positions (0°) are observed predominantly in the proteins. The most significant differences between the proteins and complete enumeration are observed for the shift *d* (Fig. 8). We see that there are sharper peaks for the protein data set compared to full enumeration of conformations for a pair of aromatic residues.

It could be assumed that the features of the β-angle, as well as shear distributions are associated with the contacts of closely spaced residues. These are contacts through one residue along the chain and interactions between residues in neighboring β-strands (bridge β-strands). However, such interactions cover only 12% of all contacts of aromatic rings. Interestingly, among the parallel contacts, the share of those close to each other in the chain or in the secondary structure is 20%. In any case, such contacts can explain no more than half of the deviations from the reference distribution.

### 3.3 Why 60° is the most favorable arrangement of aromatic residues?

As one can see, the angle between the planes has a pronounced maximum of 60°. This same maximum was observed both for the proteins and for the reference database. When enumeration is done with admissible overlapping, the maximum shifts slightly towards 90°. This fact is easy to explain.

**Fig. 9:**
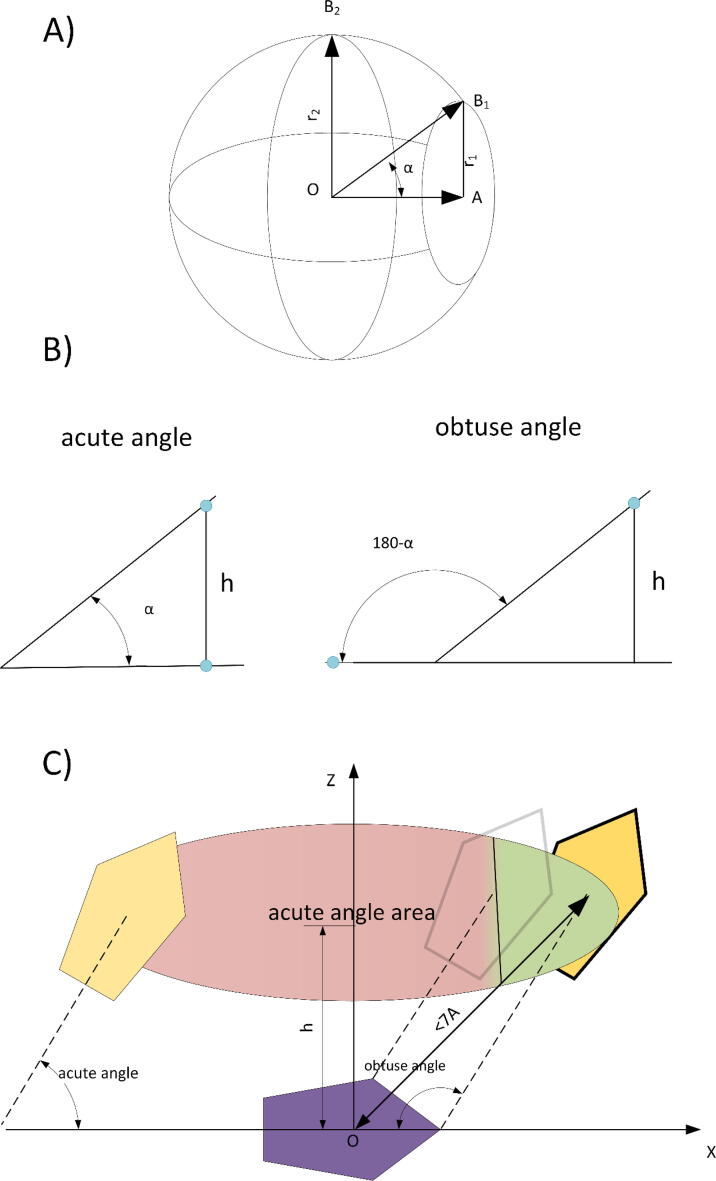
Explanation of the proportion of acute and obtuse angles. (A) The possible location of point B at a fixed angle A0B is shown schematically. The occurrence of the angle should be proportional to the circumference. It can be seen that at an angle A0B equal to 90°, the circumference is maximum. (B) The transformation of an acute angle into an obtuse one is shown when the aromatic ring moves along the X-axis. The dots show the centers of the aromatic rings, and the lines correspond to the planes in which the rings lie. (C) The same, but aromatic rings are shown. The movement parallel to the X0Y plane is considered. The area at the contact distance (less than 7 Å) forms a circle. The area of obtuse angles is shown in green, acute angles in red. One can see that the green area is smaller than the red one. (For interpretation of the references to colour in this figure legend, the reader is referred to the web version of this article.)

First, let us figure out why the distribution of the angle is uneven. To do this, consider two points in space. The first one (A) has coordinates (1,0,0). The second (B) is located on a sphere with a radius of 1 and center at the origin. Consider the angle A0B. Angle A0B is zero if points A and B coincide, and 180° if opposing, that is, B has coordinates (−1,0,0). 90° if B is located on a circle centered at (0,0,0) in a plane perpendicular to the 0X axis. The circumference is 2π. For an arbitrary angle, the points will be located on a circle with the center (cos(A0B),0,0). The circumference is 2π×sin(A0B). Thus, the probability density of the angle is proportional to its sine (sin(A0B)) (Fig. 9A). Precisely the same pattern is characteristic for the angle between the planes.

Let us choose a coordinate system so that the atoms of one of the aromatic side groups lie in the 0XY plane at z = 0 for all atoms. In this case, let the center of the aromatic group coincide with the origin (Fig. 9B, C). We will get the coordinates of the second side group by rotating and moving the first one. First, we turn it around the 0Y axis by an angle α, and then move it to a height *h* along the 0Z axis. The angle between the planes under these conditions will always be acute and equal to α if α ≤ 90°, or equal to 180° – α if α > 90°. For simplicity, we will assume that α ≤ 90°. Next, let us shift the second side group by varying Δx and Δy. In this case, the center of the second side group will always be in a plane parallel to the plane of the first aromatic group, and the distance between the planes will be equal to *h*. By changing Δy, we in no way change the angle between the planes, because we rotated around the 0Y axis. However, if we change Δx so that Δx < –h*cotg (α), then the angle between the planes will change and become equal to 180° – α, that is, the angle will change from acute to obtuse (Fig. 9).

Now, remember that we are not interested in all positions of the aromatic side groups, but only in those which are in contact. By our definition, aromatic groups are in contact if the distance between their centers is less than 7 Å. That is, $\Delta x^{2}+\Delta y^{2}<7^{2}-h^{2}$. At 0 ≤ *h* < 7, we have a circle on which the center of the second aromatic group can be located. The majority or the entire circle is occupied by centers in planes intersecting at an acute angle. The smaller is the angle α, the greater the cotg (α), and the smaller the proportion of centers per segment with angles 180° – α. In contrast, the smaller is *h* value, the closer the area of obtuse angle is approaching 50%.

### 3.4 Comparison with published results

The features of aromatic residues have been already actively studied prior to 2000, as the arrangement of these groups in proteins is central to solving the folding problem. Aromatic residues contribute to the conserved hydrophobic nucleus, to folding nuclei, to binding sites for interaction with both other proteins and with DNA. A highly conserved phenylalanine cluster forms a hydrophobic core in the villin headpiece [18]. It has been demonstrated that aromatic interactions are formed late in the protein folding process, and thus they play a role in stabilizing the folded state [19].

Parallel interaction can be energetically more favorable than interaction at an angle of 60° but only for pure pairs of aromatic residues without aliphatic–aromatic interactions in pure liquid benzene or benzene vapor. But if other hydrophobic residues are present, then this advantage is lost, as shown by our statistical analysis. As we expect, the mixture of benzene and octane in the liquid state should have the same distribution of angles and shifts as those observed in our study.

**Fig. 10:**
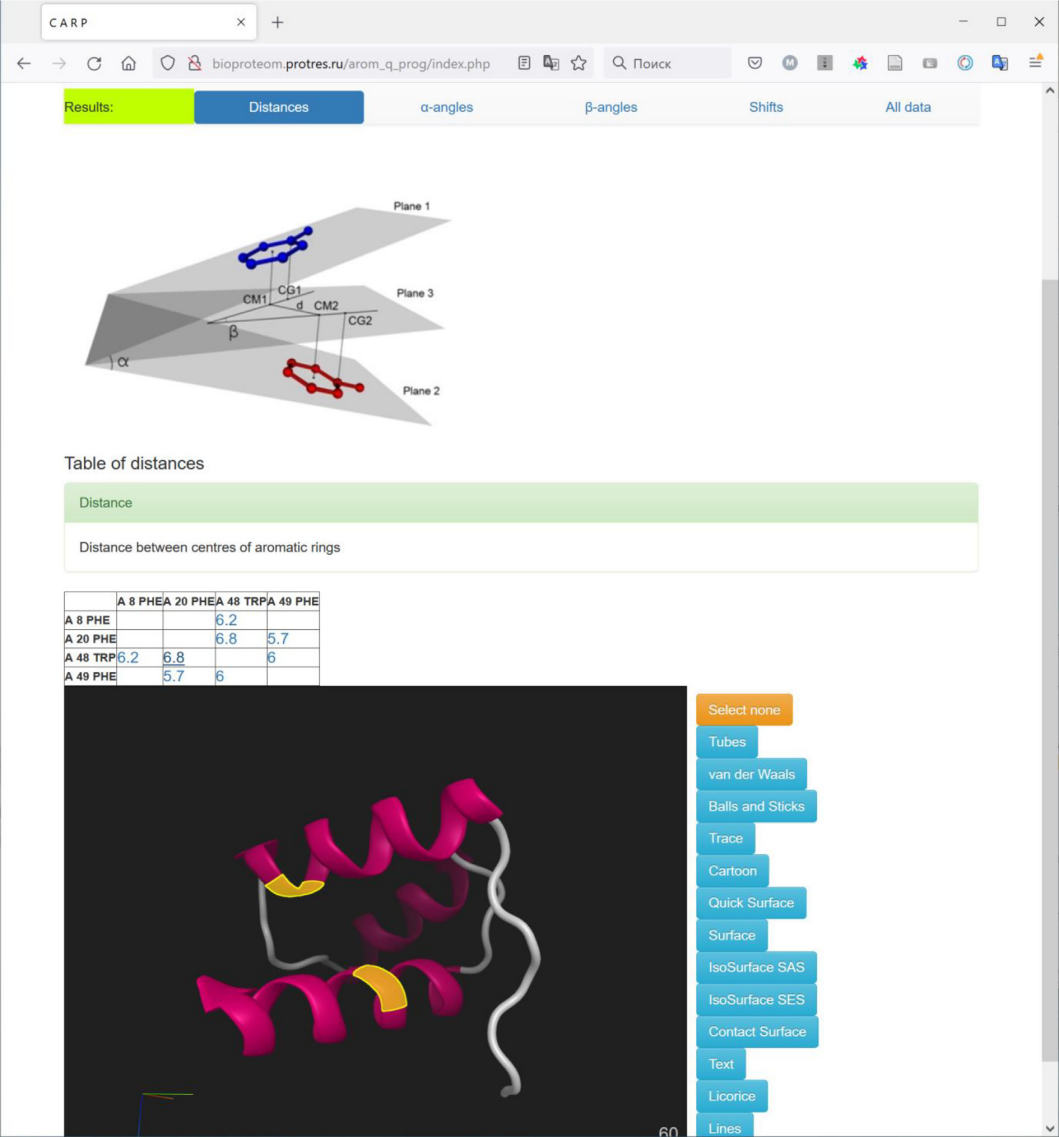
CARP server result for the protein structure 1enh and interacting aromatic residues (48 Trp and 20 Phe) are highlighted in yellow. The distance between these residues is 6.8 Å. (For interpretation of the references to colour in this figure legend, the reader is referred to the web version of this article.)

Burley and Petsko in 1985 reported that dihedral angles between phenyl rings approaching 90° were most common [5]. Then, Thornton et al. wrote about the preference for the perpendicular conformation for geometry of Phe-Phe interaction [20]. In the paper [6], the authors found that the preferred orientation is referred to as parallel shifted π-stacking. Although Fig. 10 in this work clearly indicates a high density of the cluster of points around 60°–90°, as in our case. The number of pairs we have is two orders of magnitude greater than in this paper. It turned out that the preferred orientation is closer to 60° after thorough analysis of data.

The authors have tried to compare the results of this study with best-in-class quantum mechanical calculations. The side-chain side-chain complexes from the SSI database [21] were analyzed, and only 9 His-His pairs and 15 pairs of Phe-His were found. This number of dimers is insufficient to justify whether observed conclusions are based on their real energy relationships or from other specific structural protein factors. Accuracy of dimer predictions for conventional force fields (GAFF = 2.72 kcal/mol and CGenFF = 1.3 kcal/mol) [21] is not enough to understand prevalence in the population of two times like in His-His case if it is coming from energy features [22], because their difference in the population of two times will result in small energy: (dE ∼ RT ln (2) ∼ 0.4 kcal/mol).

### 3.5 Implementation of the server

The server will provide an analysis of the packing geometry of aromatic residues. As a rule, aromatic residues are located at specific sites, and these residues keep these sites together. Using our server http://bioproteom.protres.ru/arom_q_prog/ the user can build and visualize the aromatic network, residues of which hold 3D protein structure. This site will be useful for determining the conformations of aromatic residues and detecting the possible function of aromatic stacking interactions. One example is shown in Fig. 10.

## 4 Conclusions

The present work focuses on the distribution of the relative orientation of aromatic residues in proteins when these residues are in close contact. The analysis of aromatic residues in protein structures with a level of amino acid sequences identity below 50% was carried out. Our results differ from previously published analyses of aromatic amino acids in proteins, where the preferred orientation was either the parallel shifted π-stacking [6] or T-shaped configurations [5], [20]. In our studies we found the 60° angle between aromatic residues as the most preferred arrangement in globular proteins. Parallel stacking of aromatic residues confers an energetic advantage in nucleic acids. For aromatic residues in proteins, however, the energetic benefit of parallelism, is balanced out by an entropic (steric) disadvantage. His-His pair occurs in proteins twice as often compared to the statistically expected frequency, while the His-Phe pair was found less frequently than expected. The collected and structured data were integrated into the server, which can be accessed at http://bioproteom.protres.ru/arom_q_prog/. The server can be used to analyze any protein structure in the PDB format.

## Declaration of Competing Interest

The authors declare that they have no known competing financial interests or personal relationships that could have appeared to influence the work reported in this paper.

## References

[1] Hunter C.A., Lawson K.R., Perkins J., Urch C.J. (2001). Aromatic interactions. J Chem Soc, Perkin Trans.

[2] Waters M.L. (2002). Aromatic interactions in model systems. Curr Opin Chem Biol.

[3] Meyer E.A., Castellano R.K., Diederich F. (2003). Interactions with aromatic rings in chemical and biological recognition. Angew Chem Int Ed Engl.

[4] Burley S.K., Petsko G.A. (1986). Amino-aromatic interactions in proteins. FEBS Lett.

[5] Burley S.K., Petsko G.A. (1985). Aromatic-aromatic interaction: a mechanism of protein structure stabilization. Science.

[6] McGaughey G.B., Gagné M., Rappé A.K. (1998). pi-Stacking interactions. Alive and well in proteins. J Biol Chem.

[7] Kannan N., Vishveshwara S. (2000). Aromatic clusters: a determinant of thermal stability of thermophilic proteins. Protein Eng.

[8] Bhattacharyya R., Samanta U., Chakrabarti P. (2002). Aromatic-aromatic interactions in and around alpha-helices. Protein Eng.

[9] Thomas A., Meurisse R., Charloteaux B., Brasseur R. (2002). Aromatic side-chain interactions in proteins. I. Main structural features. Proteins.

[10] Thomas A., Meurisse R., Brasseur R. (2002). Aromatic side-chain interactions in proteins. II. Near- and far-sequence Phe-X pairs. Proteins.

[11] Meurisse R., Brasseur R., Thomas A. (2003). Aromatic side-chain interactions in proteins. Near- and far-sequence His-X pairs. Biochim Biophys Acta, Proteins Proteomics.

[12] Meurisse R., Brasseur R., Thomas A. (2004). Aromatic side-chain interactions in proteins: near- and far-sequence Tyr-X pairs. Proteins.

[13] Chipot C., Jaffe R., Maigret B., Pearlman D.A., Kollman P.A. (1996). Benzene dimer: a good model for π−π interactions in proteins? A comparison between the benzene and the toluene dimers in the gas phase and in an Aqueous Solution. J Am Chem Soc.

[14] Chelli R., Gervasio F.L., Procacci P., Schettino V. (2002). Stacking and T-shape competition in aromatic-aromatic amino acid interactions. J Am Chem Soc.

[15] Balakrishnan S., Sarma S.P. (2017). Engineering aromatic-aromatic interactions to nucleate folding in intrinsically disordered regions of proteins. Biochemistry.

[16] Rahman M.M., Muhseen Z.T., Junaid M., Zhang H. (2015). The aromatic stacking interactions between proteins and their macromolecular ligands. Curr Protein Pept Sci.

[17] Lobanov M.Y., Likhachev I.V., Galzitskaya O.V. (2020). Disordered residues and patterns in the protein data bank. Molecules.

[18] Madhusudan Makwana K., Mahalakshmi R. (2015). Implications of aromatic-aromatic interactions: from protein structures to peptide models. Protein Sci.

[19] Budyak I.L., Zhuravleva A., Gierasch L.M. (2013). The role of aromatic-aromatic interactions in strand-strand stabilization of β-sheets. J Mol Biol.

[20] Thornton J.M., Singh J., Campbell S., Blundell T.L. (1988). Protein-protein recognition via side-chain interactions. Biochem Soc Trans.

[21] Burns L.A., Faver J.C., Zheng Z., Marshall M.S., Smith D.G.A., Vanommeslaeghe K. (2017). The BioFragment Database (BFDb): an open-data platform for computational chemistry analysis of noncovalent interactions. J Chem Phys.

[22] Finkelstein A.V., Badretdinov A.Y., Gutin A.M. (1995). Why do protein architectures have Boltzmann-like statistics?. Proteins.

